# Immune Thrombocytopenia Triggered by Sildenafil-Containing Rhino 69 Platinum 1000: A Case Report

**DOI:** 10.7759/cureus.87065

**Published:** 2025-06-30

**Authors:** Aishwarya Saripalli, Rupam Sharma, Kevin T Dao, Kasey Fox

**Affiliations:** 1 Internal Medicine, Kern Medical Center, Bakersfield, USA

**Keywords:** diagnosis, drug-induced immune thrombocytopenia, mechanism, rhino 69, sildenafil, treatment

## Abstract

Drug-induced immune thrombocytopenia (DITP) is a rare but serious complication resulting from exposure to certain medications, herbal products, and dietary supplements. Although various agents have been implicated, sildenafil-containing over-the-counter supplements have not previously been associated with this phenomenon.

Herein, we report a case of a 24-year-old male who presented with diffuse purpuric rash, gum bleeding, and epistaxis over three days. Laboratory testing revealed severe isolated thrombocytopenia (platelet count of 4,000/µL). Extensive workup for infectious, hepatic, autoimmune, and thrombotic etiologies was unremarkable. Upon further history, the patient disclosed use of "Rhino 69 Platinum 1000," an unregulated sexual enhancement supplement containing undeclared sildenafil over the prior two weeks. He received intravenous immunoglobulin (IVIG) and high-dose dexamethasone with a rapid platelet recovery. After discontinuing the supplement, his platelet counts normalized, and he remained symptom-free during one year of follow-up. No other cause of thrombocytopenia was identified.

This report highlights a probable case of DITP associated with the use of "Rhino 69 Platinum 1000." To our knowledge, this is a rare reported case implicating this supplement or sildenafil as a potential trigger for DITP.

## Introduction

Drug-induced immune thrombocytopenia (DITP) represents an under-recognized yet important cause of acute severe thrombocytopenia. It was first described in the nineteenth century and was noted by the onset of purpura in patients who were treated with quinine [1]. The list of culprit medications has rapidly evolved over the past centuries due to the ever-expanding pharmacopeia. This syndrome is classically associated with various agents, including antibiotics, anticonvulsants, and heparin [2]. This immune-mediated process results from drug-dependent antibodies that lead to peripheral platelet destruction, often manifesting with bleeding symptoms such as petechiae, purpura, and mucosal hemorrhage [3]. Our understanding of the pathogenesis of DITP continues to evolve. It is important to have a high degree of suspicion and identify this condition with prompt removal of offending medications [4]. However, the role of over-the-counter supplements and unregulated substances in DITP is increasingly acknowledged but remains incompletely understood.

## Case presentation

A 24-year-old male presented with complaints of a diffuse purpuric rash that began three days prior on the extremities and later involved his abdomen and back. He reported associated gum bleeding and intermittent episodes of epistaxis during the same period. He denied systemic symptoms such as fever, chills, abdominal pain, vomiting, headache, visual disturbances, confusion, decreased urine output, or dark-colored urine. His recent travel history included a visit to Mexico one month prior, but he denied any known sick contacts. The patient initially could not recall the name of an over-the-counter supplement he had purchased from a gas station two weeks earlier, but denied the use of any other medications, herbal remedies, or supplements. He had no prior episodes of similar illness or bleeding disorders.

**Figure 1:**
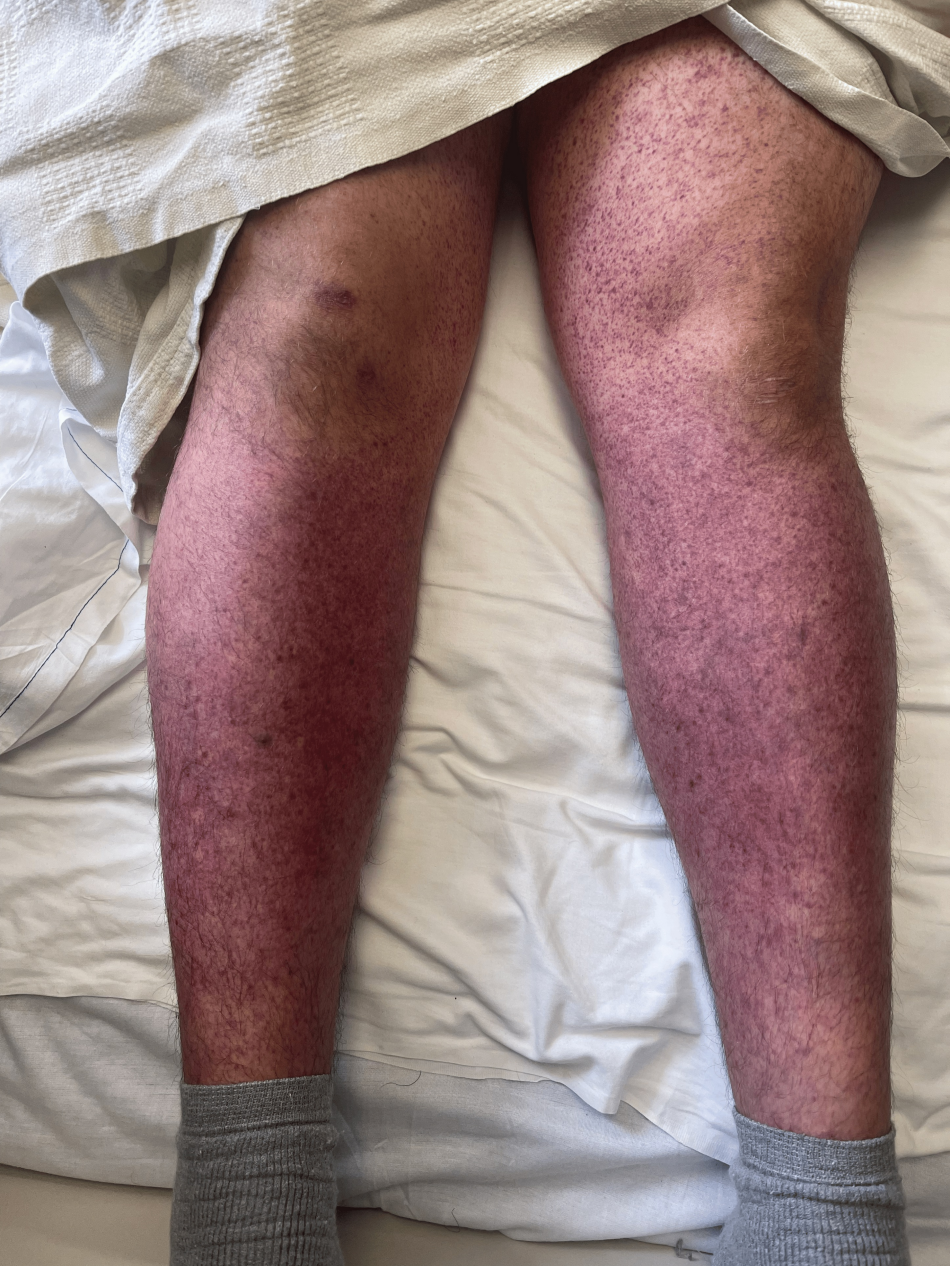
Diffuse purpuric rash

On physical examination, his vital signs were stable. Cutaneous examination revealed a diffuse purpuric rash involving the trunk and extremities (Figure 1).

Laboratory evaluation was notable for isolated severe thrombocytopenia, with a platelet count of 4,000 cells/μL (Table 1). He received two units of platelet transfusions and was started on high-dose dexamethasone. Hematology consultation recommended initiation of intravenous immunoglobulin (IVIG) alongside corticosteroids. A comprehensive workup - including evaluation for infectious etiologies such as malaria, bacteremia, and viral infections like Epstein-Barr virus (EBV) and cytomegalovirus (CMV), as well as liver disease, thrombotic microangiopathies (TTP/HUS), and autoimmune conditions - was unremarkable.

**Table 1:** Laboratory results PT: Prothrombin time; INR: International normalized ratio; PTT: Partial thromboplastin time; LDH: Lactate dehydrogenase.

Test name	Result	Reference range
Hemoglobin	14.5 g/dL	13–17 g/dL
WBC count	8.7 x 10³ cells/μL	4.5–11 x 10³ cells/μL
Platelet count	4 x 10³ cells/μL	150–450 x 10³ cells/μL
Total bilirubin	0.8 mg/dL	0–1 mg/dL
Creatinine	0.7 mg/dL	0.6–1.17 mg/dL
PT	13.7 seconds	12.1–14.2 seconds
INR	1.06	-
PTT	20.9 seconds	24.5–35 seconds
LDH	338	87–241 unit/L

Subsequently, his family provided the supplement he had ingested, identified as "Rhino 69 Platinum 1000," an over-the-counter sexual enhancement product. According to the US Food and Drug Administration (FDA) website, this supplement is known to contain undeclared sildenafil. The patient confirmed multiple uses of this product over the preceding two weeks.

Following two days of IVIG and corticosteroid therapy, his platelet count improved significantly to 66 × 10³ cells/μL. He was discharged on a tapering course of dexamethasone and was monitored regularly in the outpatient clinic over the subsequent year. During this follow-up period, his platelet counts remained within normal limits without recurrence of thrombocytopenia or bleeding symptoms.

This presentation of new-onset severe thrombocytopenia (nadir < 20 × 10³ cells/μL) with mucocutaneous bleeding occurring approximately two weeks following exposure to a supplement containing sildenafil, in the absence of other identifiable causes, strongly supports a diagnosis of DITP. The sustained resolution of thrombocytopenia after cessation of the supplement further supports this diagnosis.

## Discussion

DITP is an under-recognized clinical entity in which certain drugs or compounds - including herbal products, foods, and dietary supplements - induce thrombocytopenia by either inhibiting platelet production or promoting their peripheral destruction or clearance from the circulation [5]. Numerous agents have been implicated, including abciximab, carbamazepine, ceftriaxone, eptifibatide, heparin, ibuprofen, mirtazapine, oxaliplatin, penicillin, quinine, quinidine, rifampicin, trimethoprim-sulfamethoxazole, and vancomycin [2]. A curated list of implicated agents, based on case reports, is maintained at https://www.ouhsc.edu/platelets/ditp.html.

The pathophysiology of DITP involves drug-dependent antibodies that target platelet antigens, resulting in their destruction or accelerated clearance via the reticuloendothelial system [2]. Six mechanisms have been proposed to explain DITP [3]. The first involves hapten-dependent antibodies, where the drug acts as a hapten by covalently binding to platelet membrane proteins, thereby eliciting a drug-specific immune response. This mechanism is typically seen with agents such as penicillins and cephalosporins. The second mechanism describes drug-dependent antibodies, in which the antibodies bind to platelet membrane proteins only in the presence of the soluble drug itself. Examples include quinine, non-steroidal anti-inflammatory drugs (NSAIDs), and certain anticonvulsants. The third mechanism involves fiban-induced thrombocytopenia. In this case, drugs such as eptifibatide and tirofiban induce ligand-mediated conformational changes in glycoprotein IIb/IIIa receptors, which are then recognized by naturally occurring antibodies, leading to platelet destruction. The fourth proposed mechanism includes the formation of drug-specific antibodies that target murine components present in chimeric monoclonal antibodies, such as abciximab. The fifth mechanism is the induction of autoantibodies following drug exposure. These autoantibodies react with platelet antigens independently of the continued presence of the drug, as seen with medications like procainamide and gold compounds. Lastly, immune complex-mediated thrombocytopenia, as exemplified by heparin-induced thrombocytopenia (HIT), involves the formation of immune complexes. In this process, heparin binds to platelet factor 4, forming a complex against which antibodies are generated. These immune complexes then activate platelets via Fc receptors, leading to thrombocytopenia. Drug-dependent antibodies (DDAbs) are unusual immunoglobulins that exhibit specific binding to epitopes on platelet glycoproteins only in the presence of the sensitizing drug [3,6]. These may originate from a naturally occurring pool of weakly autoreactive antibodies that become pathologic upon drug exposure [7,8]. Typically, DDAbs emerge after one to two weeks of drug exposure and can also develop following intermittent or prolonged use [9].

DITP should be suspected in patients with new, unexplained severe thrombocytopenia, particularly if recurrent episodes follow exposure to specific foods (e.g., sesame-containing tahini [10] and tonic water with quinine [11]) or herbal teas (e.g., Jui tea [12,13]). Failure to recognize DITP may lead to unnecessary interventions such as splenectomy, especially when misdiagnosed as immune thrombocytopenia (ITP) [14]. DITP generally manifests as profound thrombocytopenia (nadir < 20,000 cells/mm³) at least one week after exposure to the inciting medication, with bleeding signs including purpura, mucosal hemorrhages like hematuria, or gastrointestinal bleeding. Rare complications include immune hemolytic anemia, neutropenia, microangiopathic hemolytic anemia, and renal failure [3]. Onset of thrombocytopenia typically occurs at least one week post-exposure, except with certain agents (e.g., abciximab, eptifibatide, and tirofiban) where symptoms may occur within hours.

The diagnosis of drug-induced thrombocytopenia is supported by the presence of five clinical criteria [9,15]. First, there should be a clear onset of thrombocytopenia following exposure to the suspected drug. Second, the platelet count typically recovers upon discontinuation of the offending agent. Third, thrombocytopenia does not recur despite the continued use of other medications, helping to isolate the causative drug. Fourth, alternative causes of thrombocytopenia must be reasonably excluded. Finally, recurrence of thrombocytopenia upon re-exposure to the suspected drug strongly supports the diagnosis, although this criterion is rarely fulfilled in clinical practice due to ethical concerns surrounding re-challenge. Confirmation via laboratory testing for DDAbs is possible but not mandatory for diagnosis [16].

Management involves prompt discontinuation of the offending agent. In cases of polypharmacy, all new drugs (within two weeks) should be discontinued [16]. Platelet counts typically recover within 4-5 drug half-lives. Platelet transfusions are often ineffective until the drug is cleared. Severe cases with bleeding manifestations warrant IVIG or corticosteroid therapy, though evidence is largely based on case reports [17,18].

To date, there are no prior reports of DITP induced by "Rhino 69 Platinum 1000" or sildenafil listed at https://www.ouhsc.edu/platelets/ditp.html. Our case suggests that sildenafil-containing supplements such as "Rhino 69 Platinum 1000" may represent a previously unrecognized cause of DITP. Although DDAb testing was not performed in this case, the temporal relationship between supplement use, severe thrombocytopenia, and sustained recovery following discontinuation strongly supports this diagnosis. We were able to successfully treat the patient with IVIg and corticosteroids, with prompt recovery of platelet count.

## Conclusions

This case highlights a probable instance of DITP triggered by the use of an over-the-counter sexual enhancement supplement, "Rhino 69 Platinum 1000," which is known to contain undeclared sildenafil. The patient’s presentation of severe thrombocytopenia with mucocutaneous bleeding, absence of alternative etiologies, and sustained recovery following discontinuation of the supplement strongly supports this diagnosis. To our knowledge, neither sildenafil nor this specific supplement has been previously reported as a cause of DITP. Given the widespread availability and unregulated nature of such products, clinicians should consider supplement-associated DITP in patients presenting with unexplained thrombocytopenia. Increased awareness and reporting of such cases are essential to broaden our understanding of the spectrum of agents capable of causing this serious and potentially life-threatening condition.
